# Prognostic relevance of Tiam1 protein expression in prostate carcinomas

**DOI:** 10.1038/sj.bjc.6603385

**Published:** 2006-09-26

**Authors:** R Engers, M Mueller, A Walter, J G Collard, R Willers, H E Gabbert

**Affiliations:** 1 Institute of Pathology, Heinrich-Heine-University, Moorenstrasse 5, Duesseldorf D-40225, Germany; 2 Department of Urology, Heinrich-Heine-University, Moorenstrasse 5, Duesseldorf D-40225, Germany; 3 The Netherlands Cancer Institute, Department of Cell Biology, Plesmanlaan 121, Amsterdam 1066 CX, The Netherlands; 4 Department of Computational Statistics, Heinrich-Heine-University, Moorenstrasse 5, Duesseldorf D-40225, Germany

**Keywords:** Tiam1, Rac, prognosis, prostate cancer, proliferation

## Abstract

The Rac-specific guanine nucleotide exchange factor, Tiam1, plays a major role in oncogenicity, tumour invasion and metastasis but its usefulness as a prognostic marker in human cancer has not been tested yet. In the present study, Tiam1 expression was analysed in benign secretory epithelium, pre-neoplastic high-grade prostatic intraepithelium neoplasia (HG-PIN) and prostate carcinomas of 60 R0-resected radical prostatectomy specimens by semiquantitative immunohistochemistry. Tiam1 proved significantly overexpressed in both HG-PIN (*P*<0.001) and prostate carcinomas (*P*<0.001) when compared to benign secretory epithelium. Strong Tiam1 overexpression (i.e. ⩾3.5-fold) in prostate carcinomas relative to the respective benign prostatic epithelium was statistically significantly associated with disease recurrence (*P*=0.016), the presence of lymph vessel invasion (*P*=0.031) and high Gleason scores (GS) (i.e. ⩾7) (*P*=0.044). Univariate analysis showed a statistically significant association of strong Tiam1 overexpression with decreased disease-free survival (DFS) (*P*=0.03). This prognostic effect of strong Tiam1 overexpression remained significant in multivariate analysis including preoperative prostate-specific antigen levels, pT stage, and GS (relative risk= 3.75, 95% confidence interval=1.06–13.16; *P*=0.04). Together, our data suggest that strong Tiam1 overexpression relative to the corresponding benign epithelial cells is a new and independent predictor of decreased DFS for patients with prostate cancer.

Prostate cancer is a leading cause of cancer death in men in North America and Western Europe. However, as compared to the high general risk of prostate cancer (one in six), the risk of death owing to prostate cancer is one in 30 (Jemal *et al*, 2003), indicating that only a fraction of cases would lead to cancer-related death if left untreated. Consequently, there is a great need for markers, which accurately predict the risk of disease progression in patients with prostate cancer and thus allow appropriate treatment planning. However, prognostic markers typically used so far, including tumour stage, Gleason score (GS), and the serum level of prostate-specific antigen (PSA) are not sufficiently precise to do so (Ross *et al*, 2003).

The guanine nucleotide exchange factor, Tiam1, specifically activates the Rho-like GTPase Rac (Michiels *et al*, 1995) and Tiam1-Rac signaling affects cell migration (Hordijk *et al*, 1997; Sander *et al*, 1998), invasion (Michiels *et al*, 1995; Keely *et al*, 1997; Engers *et al*, 2001), and metastasis (Habets *et al*, 1994) of tumour cells. Moreover, Tiam1 and Rac have been implicated in oncogenic transformation of cells. Thus, N-terminal truncation of the Tiam1 protein activates its oncogenic potential in NIH3T3 cells (van Leeuwen *et al*, 1995). Moreover, Rac is essential for Ras-induced transformation of these cells (Qiu *et al*, 1995) and in line with this, Tiam1-deficient mice are resistant to the development of Ras-induced skin tumours (Malliri *et al*, 2002). In addition, we identified several Tiam1 mutations in a significant proportion of human renal cell carcinomas, one of which proved sufficient to transform NIH3T3 cells *in vitro* (Engers *et al*, 2000). Despite these functions the potential prognostic relevance of Tiam1 expression in human tumours has not been investigated yet.

In the present study, we have analysed Tiam1 expression in benign secretory epithelium, high-grade prostatic intraepithelium neoplasia (HG-PIN), and prostate carcinomas by semiquantitative immunohistochemistry. We found that Tiam1 is significantly overexpressed in almost all prostate carcinomas and HG-PIN lesions when compared to the corresponding benign secretory epithelium. Moreover, in multivariate analysis, the extent of Tiam1 overexpression relative to the corresponding benign epithelial cells proved to be an independent predictor of decreased disease-free survival (DFS) for patients with prostate cancer.

## MATERIALS AND METHODS

### Patients

In the present study, we investigated Tiam1 expression by semiquantitative immunohistochemistry in 60 patients with R0-resected prostate cancer, who underwent radical prostatectomy at the University Hospital of Duesseldorf. Mean patient age was 65.75 years (range, 55–79 years; median, 67 years). Patients were excluded from the study if they had received neoadjuvant hormonal therapy, if surgical margins were positive, and/or if postoperative PSA values remained above 0.3 ng ml^−1^. Disease recurrence was considered in the following circumstances: (a) PSA measurement above 0.3 ng ml^−1^; (b) radiological or histological evidence of local recurrence or metastasis. Disease-free survival was calculated from the date of radical prostatectomy to the date of recurrence or last follow-up. Appropriate follow-up data were available for 53 patients. Of these, one patient died from prostate cancer, seven died from other diseases and 36 were censored. Median follow-up time of survivors was 7.2 years (range, 2–14.4 years). Mean preoperative PSA levels were 14.4±12 ng ml^−1^ (range, 1.2–60.5 ng ml^−1^). For one patient the preoperative PSA level was unknown. The study performance was approved by the ethics committee of the Heinrich-Heine-University of Duesseldorf.

### Pathological and immunohistochemical evaluation

All tumours were staged using the Tumour-Node-Metastasis (TNM) system (Sobin and Wittekind, 2002) and graded according to the system described by Gleason (1988). Moreover, all tumours were evaluated for blood vessel invasion (BVI), lymph vessel invasion (LVI), perineural invasion (PNI), and the presence of HG-PIN by the study pathologist. For immunohistochemistry representative 5-*μ*m sections of paraffin-embedded tissue specimens were subjected to heat-induced antigen retrieval and subsequently incubated with a Tiam1-specific rabbit polyclonal antibody (anti-DH, (Habets *et al*, 1994; Malliri *et al*, 2002, 2006) dilution 1 : 100) using a Ventana BenchMark immunohistochemical autostainer (Ventana Medical Systems, Munich, Germany) including the respective solutions according to the manufacturer's instructions. As a negative control, representative sections were subjected to the same immunostaining procedure except for the fact that the primary antibody was omitted. As a positive control, paraffin-embedded tumour spheroids of Tiam1-transfected renal carcinoma cells (Engers *et al*, 2001) were used. The specificity of the Tiam1 antibody used in this study has previously been demonstrated (Habets *et al*, 1994; Malliri *et al*, 2002). For quantification, the percentage of positive cells was divided into five groups: 0, 0%; 1, 1–10%; 2, 11–50%; 3, 51–80%; and 4, >80%. Moreover, a scale from 0 (no staining) to 3 (strong immunoreactivity) was assigned to staining intensity. Immunoreactive scores (range, 0–12) were calculated by multiplying percentage score of positive cells times staining intensity score (Beck *et al*, 1994). This scoring system was used, because it is well established in routine diagnostic pathology. In order to exclude potential influences of unknown differences in tissue fixation on immunohistochemical results, the extent of Tiam1 overexpression in prostate cancer relative to the corresponding normal tissue was used for statistical analyses rather than absolute immunoreactive scores. As in all cases analysed immunoreactive scores in benign secretory epithelial cells were ⩾1, the extent of Tiam1 overexpression was calculated as ratio of Tiam1 immunoreactive scores in prostate carcinoma or HG-PIN, respectively, relative to Tiam1 immunoreactive scores in the corresponding benign secretory epithelium in the same section. Slides were scored without knowledge of clinico-pathologic or disease outcome variables.

### Statistics

Tiam1 expression in benign, preneoplastic (HG-PIN), and neoplastic lesions was compared by the Wilcoxon test. Associations between Tiam1 overexpression and different clinico-pathological parameters were assessed with Fisher's exact test. The data on various biochemical and pathological parameters as well as Tiam1 expression were analysed by Cox proportional hazard method, using single variable analysis (univariate analysis) or step-wise selection (multivariate) analysis. Stratified Kaplan–Meier analyses were performed on the variables that were found to be significant in the Cox proportional hazard model. All *P*-values were two-sided. The analyses were performed with SPSS statistical software version 12.0 (SPSS GmbH, Munich, Germany).

## RESULTS

### Histological tumour characterisation

Based on histological examination 37 tumours (62%) were categorised as pT2 (organ-confined tumours) and 23 tumours (38%) as pT3 (tumours with extraprostatic extension). In one patient (2%) a single lymph node micrometastasis was observed. All patients were free of distant metastases at the time of radical prostatectomy. Gleason scores ranged from 5 to 10 with GS <7 in 11 patients (18%) and GS ⩾7 in 49 patients (82%). PNI was observed in 46 patients (77%), LVI in six patients (10%), and BVI in two patients (3%).

### Tiam1 is significantly stronger expressed in high-grade prostatic intraepithelium neoplasia lesions and prostate carcinomas than in the corresponding benign prostatic glands

**Figure 1:**
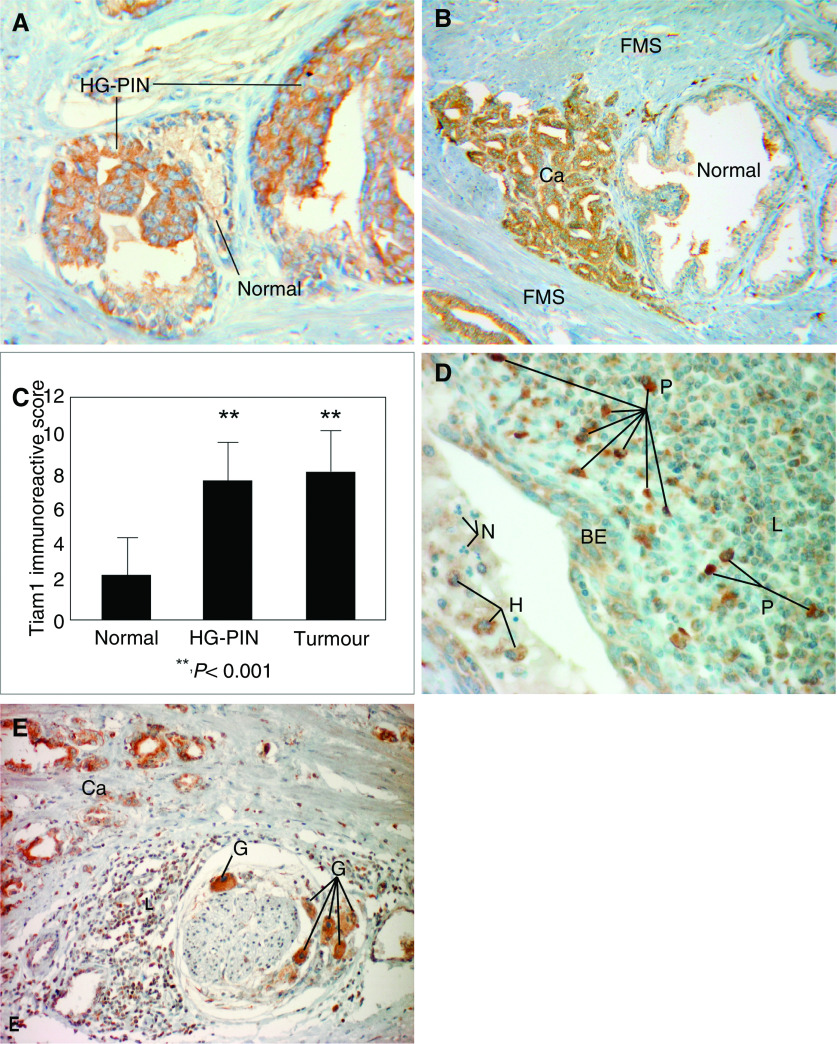
Immunohistochemical analysis of Tiam1 protein expression in human prostate cancer patients. Tiam1 expression was strongly increased in preneoplastic HG-PIN lesions (**A**) and prostate cancer (Ca) (**B**) as compared to adjacent benign secretory epithelium (Normal). Please note that fibromuscular stromal cells (FMS) were negative for Tiam1 expression. (**C**) Comparison of Tiam1 expression levels (mean±s.d.) in benign secretory epithelium (*n*=60), HG-PIN lesions (*n*=55), and prostate cancer (*n*=60) as determined by semiquantitative immunohistochemistry. Tiam1 expression levels were significantly higher in prostate cancer and HG-PIN lesions than in benign secretory epithelium (two-sided Wilcoxon test). (**D**) Tiam1 expression in inflammatory cells of prostate cancer patients. Tiam1 was strongly expressed in plasma cells (P) and weakly expressed in lymphocytes (L) and histiocytes (H). In contrast, neutrophilic granulocytes (N) proved to be negative. Moreover, Tiam1 was weakly expressed in adjacent benign epithelial cells (BE). (**E**) In addition to prostate cancer (Ca), strong Tiam1 expression was also seen in ganglion cells (G) adjacent to the prostate, whereas lymphocytes (L) exhibited only low Tiam1 expression levels. Original magnifications: (**A**) × 250; (**B**) × 100; (**D**) × 400; (**E**) × 200.

By semiquantitative immunohistochemistry significant differences in Tiam1 expression became evident between benign prostatic glands on the one hand and HG-PIN lesions and prostate cancer on the other hand. Thus, Tiam1 proved significantly stronger expressed in 54 out of 55 (98.2%) HG-PIN lesions than in the respective normal counterpart (*P*<0.001) (Figure 1A and C). Similarly 58 out of 60 (96.7%) prostate carcinomas exhibited significantly stronger Tiam1 expression levels than the respective benign secretory epithelium (*P*<0.001) (Figure 1B and C). On average, Tiam1 expression levels were 3.75-fold higher in prostate cancer and 3.6-fold higher in HG-PIN lesions, respectively, than in the corresponding benign secretory epithelial cells (Figure 1C). However, no difference was seen between Tiam1 expression in prostate cancer and HG-PIN lesions (Figure 1C). In general Tiam1 expression in prostate cancer was very homogeneous and did not differ between different Gleason patterns or different tumour foci on the same slide.

In addition to significant differences in Tiam1 expression between benign and premalignant (i.e. HG-PIN) or benign and malignant prostatic epithelial cells (i.e. cancer), respectively, we also observed differences in Tiam1 expression between normal non-epithelial cells, including inflammatory cells, fibromuscular stromal cells, and ganglion cells. Although semiquantitative analysis was not applied to these cell types, we constantly observed that amongst the inflammatory cells Tiam1 expression was markedly stronger in plasma cells than in lymphocytes and histiocytes, and that neutrophilic granulocytes appeared to be negative (Figure 1D). Moreover, Tiam1 was strongly expressed by ganglion cells (Figure 1E), whereas fibromuscular stromal cells failed to express Tiam1 (Figure 1A and B).

### Evaluating potential associations between Tiam1 protein expression in prostate cancer and different clinico-pathological factors

To analyse potential associations between the extent of Tiam1 overexpression in prostate cancer and different clinico-pathological factors (age, preoperative PSA, LVI, BVI, PNI, pT, pN, GS, and disease recurrence) Tiam1 immunoreactive ratios (e.g. Tiam1 expression levels in prostate cancer relative to those in the corresponding benign secretory epithelial cells), were dichotomised into two categories (<3.5-fold *vs* ⩾3.5-fold). The cutoff level of 3.5-fold rather than 3.75-fold (the latter of which represents the mean extent of Tiam1 overexpression in our cohort) was chosen for two reasons: (i) 3.5-fold is more convenient in terms of a potential application in routine pathology and (ii) only one patient was affected by this modification. Given a mean preoperative PSA level of 14.4 ng ml^−1^ in our cohort, preoperative PSA levels were dichotomised into <15 *vs* ⩾15 ng ml^−1^. Gleason scores was subdivided into <7 *vs* ⩾7, and pT stage into organ-confined tumours (pT2) *vs* tumours with extraprostatic extension (pT3). Other parameters such as LVI, BVI, PNI, and pN were categorised into present *vs* not present. As shown in Table 1, the extent of Tiam1 overexpression in prostate carcinoma was statistically significantly associated with the presence of LVI (*P*=0.031), GS ⩾7 (*P*=0.044) and with disease recurrence (*P*=0.016), but not with any other parameter.

### Evaluating the prognostic relevance of Tiam1 overexpression and different clinico-pathological factors in prostate cancer

#### Univariate analysis

As the patients in this cohort had variable follow-up times between 24 and 173 months, the Cox proportional hazards model and single-parameter analysis was used to determine the prognostic significance of each of the different clinico-pathological factors (i.e. age, preoperative PSA, LVI, BVI, PNI, pT, pN, and GS) as well as Tiam1 overexpression. As shown in Table 2, age, LVI, BVI, PNI, and pN did not significantly predict DFS. In contrast the extent of Tiam1 overexpression (⩾3.5-fold *vs* <3.5-fold) (*P*=0.03), preoperative PSA level (*P*=0.044), pT stage (*P*=0.006), and GS (*P*=0.001) significantly predicted decreased DFS. More specifically, the mean DFS time of patients with strong Tiam1 overexpression (⩾3.5-fold) in prostate carcinomas was 98 months (95% confidence interval (CI)=77–119), whereas the mean DFS time of patients with weak Tiam1 overexpression (<3.5-fold) was 136 months (95% CI=118–154).

#### Multivariate analysis

**Figure 2:**
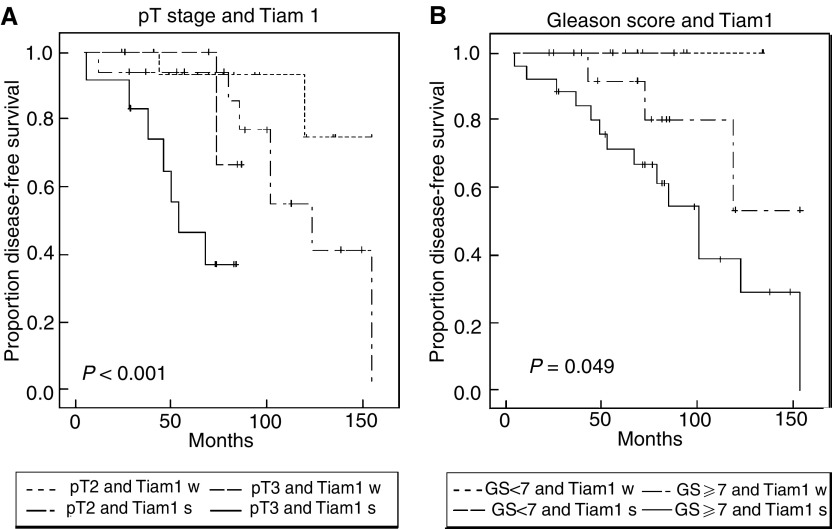
Kaplan–Meier analysis was performed after stratifying the data (**A**) as pT2 (organ-confined tumours)/pT3 (tumours with extraprostatic extension) and Tiam1 strong (s)/weak (w) and (**B**) as GS <7/⩾7 and Tiam1 strong (s)/weak (w). Statistical analysis was performed by means of the two-sided log-rank test.

To determine the smallest number of parameters that could jointly predict disease recurrence in our cohort of patients, the Cox proportional hazard model and step-wise selection analysis was used. When all parameters with significant prognostic impact in univariate analysis (i.e. preoperative PSA, pT stage, GS, and the extent of Tiam1 overexpression) were included in the model, only pT stage (*P*=0.028, hazard ratio=4.61), GS (*P*=0.019, hazard ratio=1.80), and the extent of Tiam1 overexpression (*P*=0.04, hazard ratio=3.75) reached statistical significance in predicting decreased DFS (Table 3). Moreover, including the extent of Tiam1 overexpression in the analysis further classified the pT stage and GS groups into high- and low-risk patients. To demonstrate the joint effects of Tiam1 overexpression and pT stage or Tiam1 overexpression and GS, respectively, on disease recurrence, Kaplan–Meier analysis was performed. As shown in Figure 2A, in both pT stage subgroups (pT2 and pT3) patients with strong Tiam1 overexpression (i.e. ⩾3.5-fold) had a significantly worse prognosis than patients with weak Tiam1 overexpression (i.e. <3.5-fold) (*P*<0.001). Thus, the highest probability of disease recurrence was found in patients with strong Tiam1 overexpression and pT3 stage, whereas individuals with weak Tiam1 overexpression and pT2 stage had the lowest probability of recurrence. Likewise, when the cohort was stratified into GS <7 *vs* GS ⩾7, patients with strong Tiam1 overexpression and GS ⩾7 had the highest probablility of disease recurrence, followed by patients with weak Tiam1 overexpression and GS ⩾7 (Figure 2B). The least probability of disease recurrence was found in the subgroup of GS <7. This subgroup, however, was restricted to 11 patients and no disease recurrence was observed during a median follow-up time of 72 months (range, 36–135 months). Thus, in the subgroup of GS <7 a prognostic impact of Tiam1 overexpression could not be established, at least not for the restricted number of patients and the given mean follow-up time.

### DISCUSSION

In the present study, we show for the first time that in almost all prostate carcinomas the Tiam1 protein is significantly stronger expressed than in the corresponding benign prostate epithelial cells. In addition, strong Tiam1 overexpression (i.e. ⩾3.5-fold) in prostate cancer relative to the corresponding benign epithelial cells is statistically significantly associated with decreased DFS after radical prostatectomy both in univariate analysis and in multivariate analysis, including several factors typically used to predict the prognosis of patients with prostate cancer. Therefore, our results suggest that strong Tiam1 overexpression is a new and independent predictor of disease recurrence for patients with prostate cancer and that tumours with strong Tiam1 overexpression require more aggressive treatment.

The cohort of our study is restricted to 60 patients with prostate cancer, including 53 patients with appropriate follow-up. Despite its limited size, the strength of this cohort is its restriction to R0-resected tumours, because thus disease recurrence indeed reflects tumour aggressiveness (i.e. the development of metastasis) rather than being merely the result of incomplete surgical excision of the primary tumour. Furthermore, our cohort is representative, as evidenced by the fact that well-established prognostic factors, including preoperative PSA level, pT stage, and GS, also significantly predicted disease recurrence in our study. Of these, only preoperative PSA lost its prognostic impact in multivariate analysis, when combined with the extent of Tiam1 overexpression in prostate cancer. Thus, the smallest number of parameters that could jointly predict disease recurrence included pT stage, GS, and the extent of Tiam1 overexpression. Moreover, Kaplan–Meier analysis showed that prostate cancer patients with pT3 stage and GS⩾7, respectively, could be further classified based on the extent of Tiam1 overexpression in their prostate cancer specimens to predict disease recurrence more accurately. Only in the subgroup of GS<7 a prognostic effect of the extent of Tiam1 overexpression could not be established despite a mean follow-up time of 6 years. This, however, does not exclude a prognostic relevance of the extent of Tiam1 overexpression in this subgroup. It has been shown that the overall postoperative risk of patients with GS<7 tumours to develop disease recurrence at 5 years is only about 1–2% (Epstein *et al*, 1996). Consequently, the mean follow-up time of 6 years and the number of patients (*n*=11) in the GS<7 subgroup of our cohort are not likely to be sufficient to allow the detection of new prognostic markers in this subgroup. So far, we have evaluated Tiam1 expression only in radical prostatectomy specimens, but if this marker is to be used as part of a pretreatment decision-making tool, its predictive power has to be validated in studies on biopsy specimens as well.

Increased Tiam1 expression was observed in almost all prostate carcinomas when compared to the corresponding benign epithelial cells and the distribution of Tiam1 staining is reminiscent of that reported for alpha-methyl CoA racemase (Jiang *et al*, 2001; Zhou *et al*, 2004). In contrast to alpha-methyl CoA racemase, Tiam1 expression was seen in benign glands more regularly, but expression levels were usually low and only a fraction of benign epithelial cells was positive. This suggests that similar as reported for alpha-methyl CoA racemase, strong Tiam1 expression could be used as an adjunct for the diagnosis of prostate cancer in difficult cases such as small foci in prostate needle biopsies. Nevertheless, further studies on biopsy specimens are required to finally evaluate the diagnostic potential of strong Tiam1 expression in resolving an atypical diagnosis on prostate needle biopsies.

Interestingly, significantly increased Tiam1 expression levels were not only observed in prostate cancer, but also in almost all preneoplastic HG-PIN lesions, indicating that increased Tiam1 expression occurs early in prostate carcinoma development. Although the molecular mechanism, causing increased Tiam1 expression in HG-PIN lesions and prostate cancer, has yet to be determined, there are indications that it might result at least in part from increased Wnt/*β*-catenin signaling. The Wnt/*β*-catenin signaling pathway plays a central role in colon cancer development (for review see Oving and Clevers (2002)), but in several studies aberrant activation of Wnt/*β*-catenin signaling has also been implicated in the formation of PIN-like proliferative lesions as well as in prostate cancer progression (for review see Mimeault and Batra (2006); Yardy and Brewster (2005)). Interestingly, we recently identified Tiam1 as a Wnt-responsive gene that is upregulated in intestinal and colon tumours, and by comparing tumour development in APC mutant multiple intestinal neoplasia (Min) mice expressing or lacking Tiam1, we found that Tiam1 deficiency significantly reduces the formation and growth of intestinal polyps *in vivo* (Malliri *et al*, 2006). Given these results and the established oncogenic/transforming potentials of Tiam1 and its downstream target Rac in other cell types, one might speculate that a cross-talk between Wnt/*β*-catenin and Tiam1-Rac signaling plays a major role in the development of prostate cancer, although the underlying molecular mechanism remains to be determined. Tiam1 and Rac stimulate several important signaling pathways, including the p38 mitogen-activated protein kinase (p38 MAPK), the c-Jun N-terminal kinase, and the extracellular signal-regulated kinase pathways (Coso *et al*, 1995; Zhang *et al*, 1995; Frost *et al*, 1996), which are known to regulate gene transcription. Therefore, it is conceivable that increased Tiam1 expression might induce transcription of distinct oncogenes and/or inhibit transcription of distinct tumour suppressor genes, which consequently contributes to oncogenic transformation.

In addition to its role in oncogenic transformation, Tiam1 has also been implicated in the regulation of migration, invasion, and metastasis of tumour cells (Habets *et al*, 1994; Hordijk *et al*, 1997; Adam *et al*, 2001; Engers *et al*, 2001). Interestingly, the effects of Tiam1 on migration and invasion *in vitro* may be either stimulating or inhibitory, depending on the cell-substrate used, the fact as to whether or not the formation of E-cadherin-mediated cell–cell adhesions is prevented, and the cell type studied (Sander *et al*, 1998; Engers *et al*, 2001; Price and Collard, 2001; Minard *et al*, 2004). Given these heterogeneous effects of Tiam1 on migration and invasion of epithelial cells *in vitro*, the effect of increased Tiam1 expression in a given tumour *in vivo* on DFS might be positive or negative. In prostate cancer we found that strong Tiam1 overexpression (⩾3.5-fold) relative to the corresponding benign secretory epithelium is significantly associated with decreased DFS in univariate and most importantly also in multivariate analysis. This suggests that in prostate cancer strong Tiam1 overexpression is a new and independent predictor of tumour aggressiveness. In line with this a positive correlation has been found between Tiam1 expression levels and a high tumour grade in breast cancer (Adam *et al*, 2001). In that study, however, only nine tumour samples have been analysed.

In conclusion, the present study with up to 14.4-year follow-up shows that strong Tiam1 overexpression in prostate cancer relative to the corresponding benign prostate epithelial cells correlates with aggressive disease and is an independent prognostic indicator of disease recurrence. Validation of this study involving a relatively small number of patients will establish the independent prognostic potential of strong Tiam1 overexpression in predicting disease recurrence. Moreover, it will be interesting to analyse its predictive power in further studies on biopsy specimens as well as in other tumour cell types.

## Figures and Tables

**Table 1:** Clinicopathologic features of Tiam1 overexpression in prostate cancer^a^

	**Tiam1 overexpression <3.5**	**Tiam1 overexpression ⩾3.5**	
**Factor**	**(*n*=26) No. (%)**	**(*n*=34) No. (%)**	***P*-value^b^**
*Age* (*y*)			
<65	13 (22)	13 (22)	0.435
⩾65	13 (22)	21 (35)	
			
*Preoperative PSA*^c^ (*ng ml*^*−1*^)			
<15	18 (31)	19 (32)	0.423
⩾15	8 (14)	14 (24)	
			
*Disease recurrence* d			
No	20 (38)	16 (30)	0.016*
Yes	3 (6)	14 (26)	
			
*LVI*			
No	26 (43)	28 (47)	0.031*
Yes	0 (0)	6 (10)	
			
*BVI*			
No	25 (42)	33 (55)	1.0
Yes	1 (2)	1 (2)	
			
*PNI*			
No	6 (10)	8 (13)	1.0
Yes	20 (33)	26 (43)	
			
*pT*			
pT2	18 (30)	19 (32)	0.422
pT3	8 (13)	15 (25)	
			
*pN*			
pN0	26 (43)	33 (55)	1.0
pN1	0 (0)	1 (2)	
			
*GS*			
<7	8 (13)	3 (5)	0.044*
⩾7	18 (30)	31 (52)	

BVI: blood vessel invasion; GS: Gleason score; LVI: lymph vessel invasion; PNI: perineural invasion; PSA, prostate-specific antigen; y, years.

* Statistically significant.

a Because of rounding percentages may not equal 100%.

b Fisher's exact test, two sided.

c Preoperative PSA levels were available for 59 out of 60 patients.

d Appropriate follow-up was available for 53 out of 60 patients.

**Table 2:** Univariate analysis of pre- and postoperative parameters as well as Tiam1 overexpression

**Parameter**	***χ* ^2^**	***P*-value**	**Hazard ratio**	**95% CI**
Age	1.658	0.202	1.058	0.970–1.155
Preoperative PSA	4.174	0.044*	1.035	1.001–1.070
pT	9.451	0.006*	5.805	1.657–20.339
pN	3.726	0.091	6.098	0.750–50
GS	12.685	0.001*	2.121	1.369–3.286
PNI	2.105	0.164	2.907	0.646–13.158
BVI	0.592	0.612	21.639	0.000–3126101
LVI	2.323	0.142	2.604	0.725–9.346
Tiam1 overexpression	5.531	0.030*	4	1.15–13.89

CI, confidence interval.

* Statistically significant. Cox proportional hazard model and single parameter analysis was used to determine the prognostic significance of age, preoperative PSA, LVI (+/−), BVI (+/−), PNI (+/−), pT stage (pT3/pT2), pN stage (pN1/pN0), GS, and Tiam1 overexpression (⩾3.5-fold/<3.5-fold). Age, preoperative PSA and GS were used as continuous variables.

**Table 3:** Multivariate analysis of Tiam1 overexpression with disease recurrence in patients with prostate cancer by the Cox proportional hazard method

**Variable**	*P*-value*	HR (95% CI)*
Tiam1 overexpression (⩾3.5 *vs* <3.5-fold)	0.040	3.75 (1.06–13.16)
pT (pT3 *vs* pT2)	0.028	4.61 (1.18–18.18)
GS	0.019	1.80 (1.10–2.96)
Preoperative PSA	0.134	1.03 (0.99–1.08)

CI, confidence interval; GS: Gleason score; HR, hazard ratio; PSA, prostrate-specific antigen.

* All statistical tests were two sided. Preoperative PSA and GS were used as continuous variables.
